# Nursing students' perceptions of interaction in a multiplayer virtual reality simulation: A qualitative descriptive study

**DOI:** 10.1002/nop2.2245

**Published:** 2024-07-31

**Authors:** Niina Piispanen, Elina Haavisto, Linda Hublin, Riikka Ikonen, Jaana‐Maija Koivisto

**Affiliations:** ^1^ Faculty of Social Sciences, Health Sciences Tampere University Tampere Finland; ^2^ Tampere University Hospital Tampere Finland; ^3^ Faculty of Medicine, Department of Public Health University of Helsinki Helsinki Finland

**Keywords:** interaction, multiplayer, nursing student, qualitative study, simulation, virtual reality

## Abstract

**Aim:**

To describe nursing students' perceptions of interaction in a multiplayer virtual reality (MPVR) simulation.

**Design:**

A qualitative descriptive study.

**Methods:**

Second‐semester nursing students (*n* = 24) participated in pairs in MPVR simulations and semi‐structured interviews. Data were analysed deductively and inductively.

**Results:**

Four types of interaction in a MPVR simulation were identified: interaction between the students, interaction between the student and the virtual environment (VE), interaction between the student and the virtual patient (VP), and interaction between the student and the simulation facilitator. Interaction consisted of verbal and nonverbal interaction, as well as object manipulation and movement in the VE. The reasons for interaction were to coordinate the care, to assess the VP, and to implement VPs' care.

**Conclusions:**

MPVR simulation offered nursing students an opportunity to practice nurse‐to‐nurse interaction and interaction related to nurses' collaboration, which are essential skills in nursing practice. Students were also able to interact with the VP, which can promote students' nurse–patient interaction skills. Therefore, MPVR simulations can be utilized as a platform to enhance interaction skills of future healthcare professionals, which could improve patient safety.

**Patient or Public Contribution:**

None.

## 1 BACKGROUND

Interaction is an essential component in the delivery of nursing care involving a mutual exchange of reciprocal words or actions. Strong interaction skills require competence in communication, interpersonal skills, emotional intelligence, and critical thinking. (Evans, 2016.) Interaction with technology is also an inseparable part of nursing care as nurses are the end users of technological solutions in various healthcare environments (Ruppel & Funk, 2018). Nurses who are highly skilled in their interactions with patients, families, colleagues (He et al., 2022) and technology (Ruppel & Funk, 2018) improve patient outcomes and reduce medical errors. Nursing interaction underpins many nursing functions, such as assessing patients, coordinating care, and facilitating nursing interventions (Philip et al., 2019).

Nurses' interaction skills can be enhanced with practice, but lack of nurses' confidence and difficult conversation topics with the patients can lead to a decrease in the effectiveness and quality of nursing interaction (Kerr et al., 2022). The nursing students have recognized and identified deficiencies in their interaction skills on self‐assessments, particularly related to collaboration with other healthcare professionals (Liu, 2021) and interacting with the patients (Lindqvist et al., 2022). The ability to interact efficiently and effectively is an important competence that must be acquired during nurse education (He et al., 2022).

Learning complex nursing skills, such as interaction, requires a combination of theoretical knowledge and practical training (Plotzky et al., 2021). Theoretical approaches targeted to learn interaction include techniques such as lectures, tutoring, seminars, written exams and group discussions and they aim to develop an understanding of verbal and nonverbal interaction. Practical training approaches include skills training workshops, simulations, and guided clinical practice. The goal is to acquire both verbal and nonverbal interaction skills throug authentic simulated nursing situations. (Dalcól et al., 2018.) With recent development of technology, virtual reality (VR) simulations have become a new learning method in nurse education that combines both theoretical and practical learning approaches to enhances complex nursing skills (Chen et al., 2020).

The use of multiplayer virtual reality (MPVR) simulations offers an opportunity for nursing students to practice nursing skills in a safe and controlled, simulated environment (Mäkinen et al., 2023). Numerous VR simulations have been developed to enhance learning for undergraduates. The intended learning outcomes for simulations vary from a single nursing skill to more complex and comprehensive nursing competencies like clinical reasoning and interaction. (Kamenšek, 2022.) VR simulations can be delivered using immersive technology, such as head‐mounted displays (HMDs) and haptic feedback controllers. Immersive technology provides a stronger sense of presence and immersion to its user, allowing more natural interaction compared to screen‐based VR technology. (Tao et al., 2021).

Interaction in VR can be verbal, including speech and vocalization, or nonverbal, including gestures and facial expressions (Liaw et al., 2019). Interaction with the virtual environment (VE) includes virtual object manipulation, and navigation in virtual space (Mendes et al., 2019). The literature describes four types of interactions in MPVR simulations: interaction between the players (Chen & Liou, 2022; Creutzfeldt et al., 2016), interaction between the player and the VE (Berg & Steinsbekk, 2021; Koivisto et al., 2018), interaction between the player and the virtual patient (VP) (Shorey et al., 2020; Song et al., 2023), and interaction between the player and the simulation facilitator (Mäkinen et al., 2023).

VR simulations targeted at enhancing interaction skills are still not common in nurse education, and knowledge about interaction in VR simulations is limited (Plotzky et al., 2021). The purpose of this study was to describe nursing students' perceptions of interaction in an MPVR simulation. The overall aim of this study is to develop nursing students' interaction skills using immersive technology and thus improve quality of nursing interaction and patient outcomes. The research question that guided this qualitative study was: How do nursing students describe interaction in the MPVR simulation?

## 2 METHODS

### 2.1 Design

A qualitative descriptive design with content analysis was used to conduct the study (Kim et al., 2017). The Consolidated Criteria for Reporting Qualitative Research (COREQ) checklist for focus groups and interviews (Tong et al., 2007) were used to increase the transparency of the study procedures.

### 2.2 Participants

Participants were second‐semester nursing students (*n* = 24) purposively recruited from a university of applied sciences in Finland. The MPVR simulation was an optional part of emergency care nursing course, which is part of the initial stage nursing studies. The main learning objectives of 5 ECTS credit course is to understand the legislation guiding emergency nursing, to learn about the treatment of emergency patients, to understand the importance of systematic status assessment of emergency care patients, and to plan and implement emergency patient care appropriately. In a course orientation, the researchers informed the students about the study and participation. The participating students booked a simulation session using an online booking form.

### 2.3 Ethical considerations

This study followed the ethical principles of the Finnish National Board on Research Integrity (2019). The study received ethical approval from the Human Sciences Ethics Committee of Universities of Applied Sciences in the Helsinki Metropolitan Area, and the university of applied sciences provided permission to conduct the study. MPVR simulation was a voluntary part of one nursing course for second‐semester students. The researchers informed the students about the purpose and phases of the study and explained the potential side effects of VR and safety issues during the MPVR simulation. The MPVR simulation performance was not evaluated as part of the course completion. Students were made aware of the option to leave the study at any time. Each student signed the informed consent before the simulation session and interview.

### 2.4 Procedure

The MPVR simulation used in this study was originally developed as a desktop VR simulation game (Koivisto et al., 2018). The simulation scenario was synthetized in collaboration with a multidisciplinary expert team including nurse educators, nurses, and a medical doctor. In the scenario the VP suffers from pneumonia, and the scenario story focuses on the assessment of the VP's condition using two evidence‐based tools: ABCDE approach (Perkins et al., 2021 ), and National Early Warning Score (NEWS) (McGrath et al., 2022). The content is based on international (Rhodes et al., 2017), and domestic consensus declarations (Acute Lower Respiratory Tract Infection in Adults, 2015). The simulation scenario is targeted at practicing detection of the VP's rapidly deteriorating condition and learning to use clinical reasoning.

**FIGURE 1:**
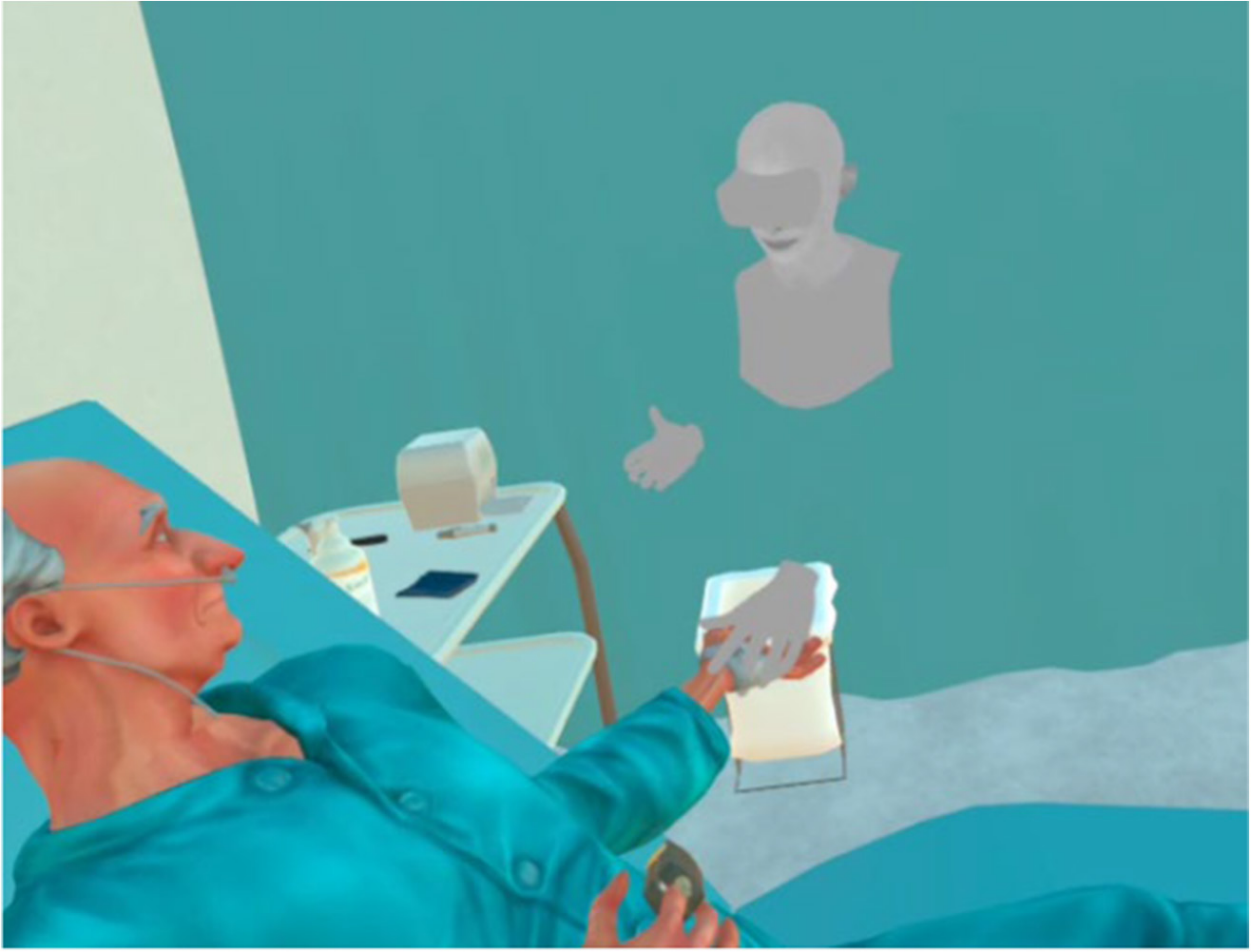
Students' avatar palpates the VP's radial artery pulse.

The MPVR simulation was delivered using HMD Oculus Quest 1 headset and controllers with haptic feedback function. Students attended the simulation in pairs to enable their mutual interaction as a dyad, and they were able to see each other's avatars during the simulation (Figure 1). Forms of interaction and implementation are presented in Data S1.

Before entering the simulation, the participants familiarized themselves with the VR environment and practiced using the controllers. Thereafter they entered the simulation scenario together. The researchers supervised the simulations to ensure participants' safety and instructed them on technical issues, but not on nursing‐related matters. Simulation sessions lasted from 15 to 35 min.

**FIGURE 2:**
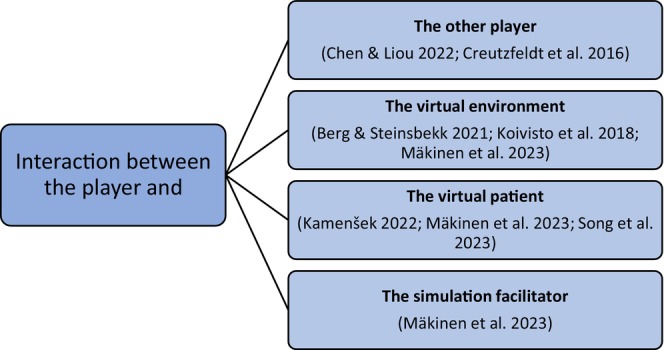
Interview framework.

After the simulation, students completed a background questionnaire. Thereafter, two researchers conducted semi‐structured interviews face to face in student pairs using open‐ended questions (McGrath et al., 2019). The interviews were guided by a semi‐structured interview framework constructed from the four types of interaction in MPVR simulations. During the interviews the participants were asked to describe their experiences of interaction around all four different types of interaction (Figure 2). They were also asked to describe the content of interaction in detail with each party of the interaction.

A total of 12 interviews lasting from 31 to 71 min were audio‐recorded. After the ninth interview, the data reached saturation, but interviews were continued to allow participation by all students who volunteered. The audio‐recordings were transcribed verbatim in Finnish.

### 2.5 Data analysis

Qualitative deductive and inductive content analysis was conducted to analyse the data. First, one researcher read through the data several times to get a full understanding of the content. Second, a categorization matrix was developed deductively (Elo & Kyngäs, 2008) based on previous research about the basic types of interaction in the MPVR simulations (Figure 2). Third, all meaningful expressions from the data were identified, reduced, and entered into the categorization matrix (Elo & Kyngäs, 2008) under one of the four main types of interaction. Lastly, the expressions were inductively analysed within each main category, and synthetized into 15 subcategories. The subcategories were named to describe the content. (Lindgren et al., 2020.) All researchers critically appraised the entire analysis, and changes were made as needed to reflect consensus.

## 3 RESULTS

### 3.1 Participant characteristics

Most of the participants were female (*n* = 18), and aged 25 years or younger (*n* = 20). The full participant characteristics data are presented in Table 1.

**TABLE 1:** Participant characteristics (*n* = 24).

CHARACTERISTIC	*n*
Age	
25 years or younger	20
26 to 30 years	2
31 years or older	2
Gender	
Female	18
Male	6
Educational backround	
Baccalaureate	12
Practical nurse degree	2
Other vocational degree	6
Baccalaureate and vocational degree	4
Nursing experience	
None	16
Less than one year	4
1–2 years	4
Previous experience of VR in the last year	
Yes	11
No	13

### 3.2 Nursing students' perceptions of types of interaction in the MPVR simulation

**FIGURE 3:**
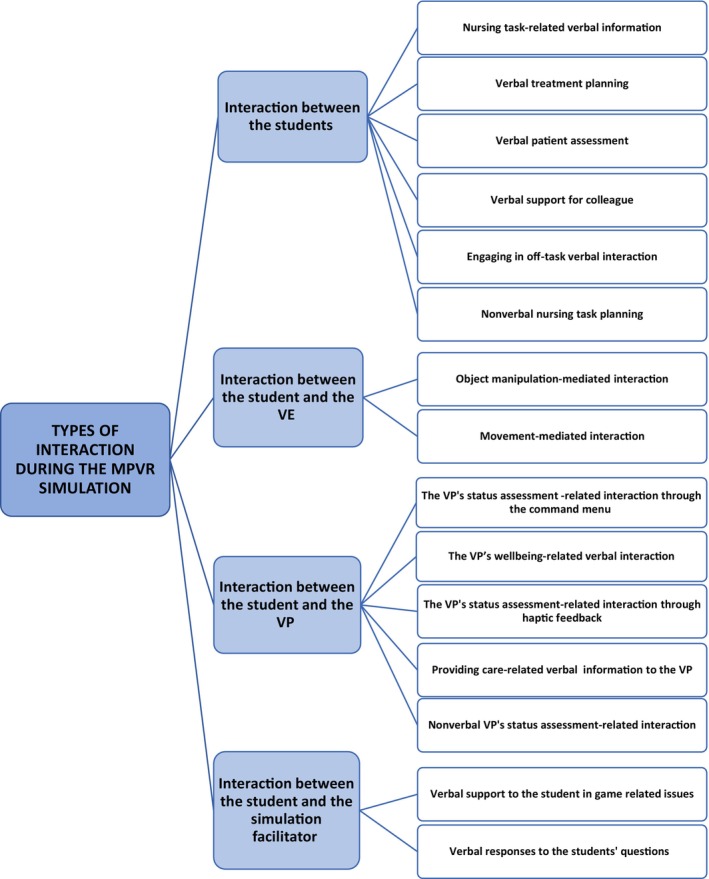
Nursing students' perceptions of types of interaction in the MPVR simulation.

Based on the analysis, nursing students emphasized four types of interaction in the MPVR simulation that aligned well with the four types identified in the literature: interaction between the students, interaction between the student and the VE, interaction between the student and the VP, and interaction between the student and the simulation facilitator (Figure 3).

### 3.3 Interaction between the students

Interaction between the students included six subcategories: nursing task‐related verbal information, verbal treatment planning, verbal patient assessment, verbal support for collegue, engaging in off‐task verbal interaction, and nonverbal nursing task planning.

Nursing task‐related verbal information consisted of informing the co‐player (the nursing student partner in the simulation) about the ongoing work task, negotiating distribution of the work tasks, and discussing the current situation to maintain mutual situational awareness. Some of the students stated they were not able to locate one another's avatars all the time, so they had to share information about the ongoing task by describing it aloud. Some stated that the work tasks were easier to distribute by talking aloud from the very beginning of the simulation. Some participants found it was important to discuss a rapidly changing patient situation immediately to maintain situational awareness.

‘*The partner occasionally tells you what they are doing and what was, for example, the blood pressure result, and as a result you maintain situational awareness*.’ P19

Verbal treatment planning included negotiating about the treatment plan, confirming the co‐player's suggestions regarding patient care, and justifying their opinions regarding clinical decisions. Some students stated they worked together equally with the co‐player to plan the VP's care during the MPVR simulation. Some stated that they had to justify their opinions to one another and define what their decisions were based on.

‘… *you asked me why I do not put an oxygen mask on the patient or something, and I answered because the oxygen saturation is this and that*.’ P24

Verbal patient assessment included discussing the physiological measurements from the patient, discussing issues related to the patient's condition, and assessing the patient's condition by discussing his NEWS. Some students stated that discussing the physiological measurements of the VP together helped them make decisions and prioritize. They also discussed the VP's NEWS together to assess his clinical condition collaboratively.

‘…*I counted the NEWS and thought*, *oh*, *his scores are that high and he (the patient) is dying. We also discussed that through together*. P3

Verbal support for colleague included advising the co‐player to perform a treatment procedure, advising the co‐player locating the medical equipment, and giving feedback to the co‐player. Some of the students said they did not give any actual feedback to one another, but they felt like they provided a kind of feedback by helping one another or complying with the other's suggestions.

‘*Maybe it (feedback) was more like saying yes, good, and yeah let's do that, or wait I'll help you*.’ P15

Engaging in off‐task verbal interaction consisted of joking with the co‐player and laughing about the difficulties faced during the simulation. Some students said they would never joke or laugh in a real patient situation, but with the VP it was easy, because he was not a real human.

‘*Maybe we laughed a little at the mistakes we made, something we might not do with a real patient*.’ P20

Nonverbal nursing task planning included distributing the work tasks based on gestures of the co‐player and handing over medical equipment to the co‐player. The students were able to see each other's avatars, which made it easier to perceive what the co‐player was doing. Some students found the ability to hand over equipment to the other useful, as they could focus on patient care instead of moving around looking for equipment. Conversely, some of them found handing over equipment difficult because there was visual inaccuracy.

‘*I saw you having that item in your hand; it helped as I saw what the other person was doing*.’ P5

### 3.4 Interaction between the student and the VE

Interaction between the student and the VE included two subcategories: object manipulation‐mediated interaction, and movement‐mediated interaction.

Object manipulation‐mediated interaction consisted of marking the VP's NEWS on the scoreboard, dispensing hand sanitizer, adjusting the patient's bed, measuring the patient's vital signs with various medical equipment, adjusting the oxygen flow meter, and browsing the patient report. Some students found the interactive NEWS scoreboard useful because they were able to mark the VP's scores on the board instead of memorizing them. The authenticity in use of the medical equipment surprised the students because equipment such as thermometer looked like and functioned as in the real world.

‘*As I took the VP's temperature, I really had to hold the thermometer in his ear*.’ P2

Movement‐mediated interaction included moving around in the patient room by walking in the game playing area and teleportation. Some students stated that moving in the VE was smooth, and they felt they were able to freely move around. Conversely, some students found it difficult to move around on foot because they were disturbed by the visible security boundaries of the playing area. Some students said that teleportation was an easy and smooth way to move around in the VE.

‘*If I wanted to pick up an item in the VE, I just walked to it, pressed the trigger and picked it up*.’ P23

### 3.5 Interaction between the student and the VP

Interaction between the student and the VP consisted of five subcategories: the VP's status assessment‐related interaction through the command menu, the patient's wellbeing‐related verbal interaction, the VP's status assessment‐related interaction through haptic feedback, providing care‐related verbal information to the VP, and nonverbal VP's status assessment‐related interaction.

The VP's status assessment‐related interaction through the command menu included asking the VP about his condition, interviewing the VP about his response to treatment, and prompting the VP to move his limbs. Some students stated that there was an appropriate number of questions in the command menu to be asked from the VP. Some students found it difficult to communicate through the command menu, and they stated that they would rather ask the questions aloud. Some stated that the command menu lacked alternatives, such as asking about VP's pain or thirst.

‘*He was a tricky patient, I had to press a button if I wanted him to respond and say something*.’ P5

The patient's verbal wellbeing‐related interaction included the VP talking about having difficulties with breathing, saying he did not feel nauseous, and telling the player he was not feeling well. Some students stated that the VP could have been more interactive, and they found the VP's answers were always the same.

‘*He (patient) did not really say anything more than that he is feeling bad, not nauseous, and having difficulties breathing*.’ P5

*The VP's status assessment‐related interaction through haptic feedback included palpating the VP's wrist to measure radial* artery pulsation and grabbing the VP's hands to assess the grip strength. Students were also able to estimate the heart rate and assess the symmetry of the VP's grip strength through the haptic feedback of the controllers.

‘*When I took the wrist pulse, I said it is steady but fast*.’ P2

Providing care‐related verbal information to the VP consisted of introducing oneself to the VP and informing the VP about the ongoing treatment procedures. Some students stated that they forgot to introduce themselves to the VP because they did not consider him as a real patient.

‘*First thing is to introduce yourself to the patient, such a basic thing, but in this situation, it somehow did not even cross my mind*.’ P7

Nonverbal VP's status assessment‐ related interaction included interpreting the gestures and interpreting the facial expressions in the treatment situation. Some of the students stated that they could see the patient was having difficulty breathing. Other students said that the VP's gestures did not resemble the behaviour of a septic patient and that his appearance did not correspond to the severity of his condition.

‘*No alarm bells rang, grandpa just smiled on the bed and seemed well*.’ P22

### 3.6 Interaction between the student and the simulation facilitator

Interaction between the student and the simulation facilitator consisted of two subcategories: verbal support to the student in game related issues, and verbal responses to the students' questions.

Verbal support to the student in game related issues included advising the player about how the game functioned and the controllers worked. Some students found the provided instructions to be clear, and they felt they had received sufficient technical guidance.

‘*In my opinion, the technical guidance was completely sufficient*.’P21

Verbal responses to the students' questions consisted of explanations regarding interacting with the VP, questions regarding game functions, and guiding the player in matters related to how to progress in the MPVR simulation. Some students stated the simulation would have been hard to complete successfully without the opportunity to seek guidance from the simulation facilitator during the simulation session.

‘*It would have been hard if you could not ask instructions during it (simulation) like I did*…’ P12

## 4 DISCUSSION

The purpose of this study was to describe nursing students' perceptions of interaction in the MPVR simulation in nurse education. The results reinforced the previous findings from the literature about the four types of interaction in the MPVR simulations. They were interaction between the students, interaction between the student and the VE, interaction between the student and the VP, and interaction between the student and the simulation facilitator.

The first main category, interaction between the students, emphasized students' mutual interaction during the MPVR simulation. In this study, participating in the simulation as pairs provided nursing students with an opportunity to practice nurse‐to‐nurse interaction. They said their mutual interaction in the simulation was targeted to assess the patient, coordinate his care, and facilitate nursing interventions. This evidence aligns with findings from Philip et al. (2019) that interaction between the players during MPVR simulation mimics real‐world nursing interactions and suggests that MPVR activities prepare nursing students to interact effectively with nursing colleagues. In this study, the students expressed that nonverbal interaction between the students was limited to task planning, and the students' mutual interaction did not include facial expressions at all. According to Shorey et al. (2020), nonverbal gestures and facial expressions could be added to enhance the realism of interaction in VR simulations.

The second main category, interaction between the student and the VE, was mediated through object‐manipulation and movement. Students reported that they were able to interact with the VE and they found it quite realistic. The ability to use the medical equipment to examine the VP increased the sense of interactivity. This finding is consistent with Mäkinen et al. (2023) who found that the interactivity of the VE reinforces students' sense of participation during the VR simulation. Finding also aligns to Tao et al. (2021), who discovered that the use of highly immersive technology provides a stronger sense of presence and immersion to the students allowing more natural interaction during the MPVR simulation. In this study, teleportation was perceived as an effortless way to move in the VE whereas moving around on foot was inconvenient for some because the playing area was restricted with the security boundaries. According to Liaw et al. (2019), problems like this can weaken the interaction with the VE, because they hinder concentration and cause frustration. Creutzfeldt et al. (2016) stated that physical actions in VR can cause challenges and uncertainty among students with less VR experience. Therefore, they may need more pre‐simulation instruction and opportunities to practice movement in the VE, which should be considered as VR simulations are implemented as a learning method (Creutzfeldt 2016).

Opposing perceptions emerged in the third main category, *interaction between the student and the VP*. Some students felt interaction with the VP was easy and clear, while others found it clumsy and difficult. Koivisto et al. (2018) discovered that students favour more interactive and realistic VPs in VR simulations, and according to Shorey et al. (2020), poor interaction with the VP can interrupt the flow of the simulation. The interpretation by students of VP's nonverbal communication was inconsistent in this study. Some students said that it was easy to interpret the VP's body language whereas other students felt that it did not resemble the severity of his situation. Findings by Shorey et al. (2020) suggested that VP's poor expression of his situation can lead to lack of realism and decrease the sense of urgency in VP's care.

The last main category was the interaction between the student and the simulation facilitator. The simulation facilitator had an essential role as technical support during the MPVR simulation. Findings related to the importance of facilitators' role as technical support provider are consistent with the previous findings of Mäkinen et al. (2023). In addition, Song et al. (2023) also emphasized the importance of technical guidance, especially for students who have no previous VR simulation experience (Song et al., 2023). The findings of this study indicate that the simulation facilitator needs to be familiar with the VR technology to be able to deliver adequate technical support to students before and during a VR simulation.

### 4.1 Strengths and limitations

There were certain strengths and limitations in this study. The students were from one university of applied sciences, and the results might not be transferrable to other settings. Two researchers carried out the interviews separately, which may have caused bias. However, the same interview framework was used to ensure that the interviews were conducted as identically as possible. The concept of interaction in VR might have been difficult for students to understand fully, which could have made it difficult to answer the interview questions. To avoid this, the concept of interaction was explained to the students before the interview. To enhance the trustworthiness of this study, researchers reflected on their social positions that could have had influence on the study and their ability to remain objective (Adler, 2022). The context and participant characteristics were described to improve transferability (Santiago‐Delafosse et al., 2016), and data saturation enhanced credibility of the study (Stenfors et al., 2020). The analysis process was revised, and the results were approved by the research group to enhance credibility (Dyar, 2022), and dependability (Granehaim et al., 2017). The checklist for Researchers Attempting to Improve the Trustworthiness of a Content Analysis Study was used to improve trustworthiness of the analysis (Elo et al., 2014).

## 5 CONCLUSION

In summary, MPVR simulation offered nursing students an opportunity to learn nurse‐to‐nurse interaction, and interaction related to nurses' collaboration, which are essential skills in nursing practice. Students were also able to interact with the VP, which can promote students' nurse–patient interaction skills. Therefore, MPVR simulations can be utilized as a platform to enhance interaction skills of future healthcare professionals, which could improve patient safety. Insight into how interaction occurs in MPVR simulations from the student perspective will help faculty develop effective approaches to enhancing students' interaction skills using immersive technology. Future research should target to investigate the factors that influence interaction in MPVR simulations and the potential learning outcomes. In the future it is also necessary to investigate the role of technology in the interaction learning and to compare MPVR simulations to traditional interaction learning methods.

## AUTHOR CONTRIBUTIONS

Writing the manuscript: NP. Study design: JM‐K, EH. Data collection: NP, LH. Data analysis and interpretation: NP, EH, LH, RI, J‐MK. Critical revision of the article: EH, LH, RI, J‐MK. Final approval of the version to be published: NP, EH, LH, RI, J‐MK.

## FUNDING INFORMATION

This research did not receive any specific grant from funding agencies in the public, commercial, or not‐for‐profit sectors.

## CONFLICT OF INTEREST STATEMENT

N/A.

## ETHICS STATEMENT

The Human Sciences Ethics Committee of Universities of Applied Sciences in the Helsinki Metropolitan Area.

## Supporting information


Data S1.

## Data Availability

Authors elect to not share data due to ethical restrictions.
