# Supplementary material for: Nursing students' perceptions of interaction in a multiplayer virtual reality simulation: A qualitative descriptive study
Source: Nurs Open. 2024 Jul 31;11(8):e2245. doi: 10.1002/nop2.2245 (PMC11290554; doi:10.1002/nop2.2245)
Supplement: Supplementary file 1 — Data S1. [file NOP2-11-e2245-s001.docx]

**Supplementary digital material**:

The forms, examples, and implementation of interaction in the MPVR simulation using Oculus Quest 1 HDM headset and controllers (Koivisto et al., 2018; Oculus Quest 1 manual, Nd)

| **Form of interaction** | **An example** | **Implementation** |
| --- | --- | --- |
| ***Reading*** | Reading text or numbers in the patient report, from patient monitor or NEWS-table | Text, numbers, and symbols in fixed position in VR space, longer texts are scrolled by pressing index trigger of the controller and moving hand up or down. |
| ***Looking*** | Searching for medical supplies or inspecting patients skin color | Optics and display of the headset: Fresnel-lenses, single fast switch LCD binocular-display, resolution 1832x1920 pixels per eye. |
| ***Navigation by teleportation*** | Moving around by teleportation using the controller | The desired teleportation location is pointed using 6-DoF Outside-in hand tracking, and teleportation is done using the index trigger of the controller. |
| ***Locomotion*** | Leaning on or turning around in VR by moving physically | The headset and the controllers include sensors to track physical movement which is translated into VR movement. |
| ***Changing the viewing angle*** | Reversing the direction of the first-person eyesight using the controller | The viewing angle is changed using the joystick of the controller or by head movement. |
| ***Command menu*** | Interviewing the VP through command menu located in the wall behind VP's bed | Command menu becomes visible by pressing face button, and the desired box can be selected by pointing it with hand and pressing the index trigger of the controller to activate the selected function. |
| ***Gestures*** | Player's avatar extending hands to the VP | Gestures are created using hand movement traced by 6 DoF Outside-in hand tracking, which converts physical movement into VR movement (both controllers include hand tracking ring). |
| ***Postures*** | Player's avatar leaning on towards the VP | Postures are created by physical movements that are tracked by a sensor integrated into headset and controllers, which is converted into VR movement. |
| ***Touching or palpating*** | Palpating the radial pulse from VP | The player can palpate the patient's pulse or hand clamping forces and receive haptic feedback through the controllers by placing the hand at the palpation site or extending hands towards the patient. |
| ***Grabbing objects*** | Player’s avatar grabbing the oxygen mask | Objects can be grabbed and held by extending the hand towards the object and pressing the middle finger trigger of the controller to activate the grabbing function. |
| ***Handing over the objects*** | Handing over and placing an oxygen mask on the VP | Objects can be handed over by first grabbing the object by pressing the trigger of the middle finger, after which the object is transported to the desired location (by teleportation, walking or turning), and released by letting go of the trigger. |
| ***Vocalization*** | Groaning of the VP | Audio is transmitted via a microphone, and stereo speakers integrated into VR headsets. |
| ***Speech*** | The player speaking aloud the results of vital signs measurements | Audio is transmitted via a microphone, and stereo speakers integrated into VR headsets. |

**References:**

Koivisto J-M, Haavisto E, Niemi H, Haho P, Nylund S, Multisilta J. (2018). Design principles for simulation games for learning clinical reasoning: A design-based research approach. *Nurse Education Today*, 60, 114–120.

Oculus Quest 1 manual. Nd.
